# The roles of ERAS during cell lineage specification of mouse early embryonic development

**DOI:** 10.1098/rsob.150092

**Published:** 2015-08-12

**Authors:** Zhen-Ao Zhao, Yang Yu, Huai-Xiao Ma, Xiao-Xiao Wang, Xukun Lu, Yanhua Zhai, Xiaoxin Zhang, Haibin Wang, Lei Li

**Affiliations:** 1 State Key Laboratory of Stem Cell and Reproductive Biology, Institute of Zoology, Chinese Academy of Sciences, Beijing 100101, People's Republic of China; 2 Institute of Zoology, University of Chinese Academy of Sciences, Beijing 100049, People's Republic of China; 3 Institute for Cardiovascular Science and Department of Cardiovascular Surgery of the First Affiliated Hospital, Soochow University, Suzhou 215000, People's Republic of China

**Keywords:** *Eras*, primitive streak, mesoderm, embryonic stem cells, cell lineage

## Abstract

*Eras* encodes a Ras-like GTPase protein that was originally identified as an embryonic stem cell-specific Ras. ERAS has been known to be required for the growth of embryonic stem cells and stimulates somatic cell reprogramming, suggesting its roles on mouse early embryonic development. We now report a dynamic expression pattern of *Eras* during mouse peri-implantation development: its expression increases at the blastocyst stage, and specifically decreases in E7.5 mesoderm. In accordance with its expression pattern, the increased expression of *Eras* promotes cell proliferation through controlling AKT activation and the commitment from ground to primed state through ERK activation in mouse embryonic stem cells; and the reduced expression of *Eras* facilitates primitive streak and mesoderm formation through AKT inhibition during gastrulation. The expression of *Eras* is finely regulated to match its roles in mouse early embryonic development during which *Eras* expression is negatively regulated by the *β*-catenin pathway. Thus, beyond its well-known role on cell proliferation, ERAS may also play important roles in cell lineage specification during mouse early embryonic development.

## 1. Introduction

Totipotent mammalian zygote undergoes several cleavage divisions to form the blastocyst composed of the trophoblast and inner cell mass which continuously segregates into the hypoblast and the epiblast [1]. Because the epiblast can give rise to all cell lineages of the fetus and individual epiblast cells can commit into three germ layers when they are injected into blastocyst, pre-implantation epiblast is thought to be the developmental ground state [2]. After implantation, the embryo dramatically increases its cell number dependent on a rapid burst of pluripotent cell proliferation with average generation times of approximately 4.5–8.0 h [3]; at the same time, the embryo enters the primed state and initiates cell lineage specification through gastrulation, during which three primary germ layers are established [2]. Numerous cell-cycle regulators and pluripotent transcription factors have been shown to be involved in the rapid proliferation in mouse early embryos [4]. However, little is known about cell lineage specification during this process.

Mouse early embryos include a population of pluripotent cells which were cultured with fibroblast feeder cells *in vitro* to form embryonic stem cells (ESCs) [5,6]. Although the precise origin and identity of ESCs *in vivo* has long been debated [7], recent research showed that mouse ground state ESCs closely resemble the cells in pre-implantation epiblast of E4.5 embryos [8]. The gene expression pattern of ESCs is heterogeneous when they are cultured in serum and leukaemia inhibitory factor (LIF) without feeders [9]; however, their gene expression pattern becomes homogeneous when they are maintained with the inhibitors MEK and GSK3 (2i) [10]. Considering their stability, homogeneity and equipotency, ESCs in the 2i condition are thought to be an early epiblast-like ground state for embryonic development [11]. Thus, the pluripotency is proposed as two phases: naive and primed state [2]. Mouse ESCs can propagate without ERK signalling; however, the independence of ESCs on ERK signalling is lost in post-implantation egg cylinder cells [8]. The inhibition of ERK signalling is critical for maintaining ESCs in the ground state [12–14], and the activation of ERK1/2 by FGF4 is important for naive ESCs to exit from self-renewal [15]. Other factors, FGFR, SHP2 and GRB2, have also been shown to regulate ERK activity at different molecular levels in ESCs [15–17]. However, the detailed regulation of ESC pluripotency from naive into primed state still needs to be defined.

Gastrulation is a critical process of embryogenesis, through which three primary germ layers are established. *Nodal* heterozygous mouse embryos undergo gastrulation, but then display abnormalities in positioning of the antero-posterior axis, midline patterning and left–right asymmetric development. Furthermore, *Nodal* null mutations show blocked gastrulation and mesoderm formation [18]. In *Wnt3* knockout embryos, egg cylinder develops normally, but the embryos do not form primitive streak (PS), mesoderm or node [19]. *Bmp4* homozygous mutant embryos die between E6.5 and E9.5, and show little or no mesoderm differentiation [20]. Thus *Nodal*, *Wnt* and *Bmp4* signalling pathways play critical roles in cell specification of three primary germ layers during mouse gastrulation [18–21]. In addition, E-cadherin is decreased during gastrulation and has been shown to function through epithelial–mesenchymal transitions [22–25], implying the important roles of downregulated genes during this process. Currently, the roles of downregulated genes during gastrulation are largely unclear.

*Eras*, encoding a Ras-like GTPase protein, was originally identified as *Ecat5* (ES cell-associated transcripts) by analysing the mouse EST databases and is involved in tumourigenicity of mouse ESCs [26]. It has been shown that ERAS binds to phosphatidylinositol 3 kinase (PI3K; p110*δ*), instead of Raf1, to phosphorylate AKT to promote the growth of ESCs [26] and that increased ERAS–AKT–FOXO1 signalling stimulates the efficiency of somatic cell reprogramming [27], suggesting its role in cell proliferation of mouse early embryonic development [28]. Human *ERAS* was initially characterized as a homologue of mouse *Eras* [26]; later research revealed the *ERAS* gene is absent in human ESCs and concluded that *ERAS* exists as a pseudogene in humans. Several groups reported that human *ERAS* is involved in human tumourigenesis. The full-length transcript and protein were recently reported to be expressed in several gastric cancer cell lines and in some human gastric cancer tissues [29–32]. Currently, the exact role of *Eras* in mouse and human early embryonic development is still largely unknown.

In this study, we find *Eras* expression increases at the blastocyst stage, and decreases specifically in E7.5 mesoderm. The enhanced expression of *Eras* stimulates cell proliferation through AKT activation and accelerates ESC commitment from ground to primed state through ERK activation. The reduction of *Eras* facilitates PS and mesoderm differentiation through AKT inhibition during germ layer specification. Furthermore, we demonstrate the expression of *Eras* is negatively regulated by *β*-catenin signal in ESCs, epiblast stem cells (EpiSCs) and mouse early embryos.

## 2. Results

### 2.1. The dynamic pattern of *Eras* expression during mouse germ layer specification

**Figure 1.**
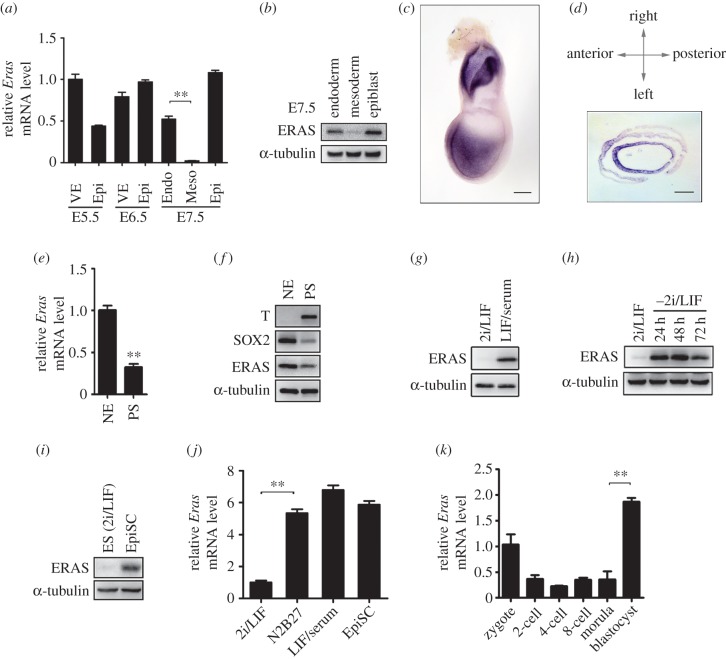
The expression of *Eras* in mouse early embryos and embryonic stem cells. (*a*) Real-time RT-PCR analysis of *Eras* mRNA in E5.5–7.5 germ layers (one-way ANOVA; **p < 0.01). VE, visceral endoderm; Epi, epiblast; Endo, endoderm; Meso, mesoderm. (*b*) Western blot analysis of ERAS protein in E7.5 germ layers. (*c*) Whole-mount *in situ* hybridization of *Eras* mRNA in E7.5 mouse embryos. (*d*) Cross-section of E7.5 embryonic region after whole-mount *in situ* hybridization. Scale bars in (*c*) and (*d*), 100 µm. (*e,f*) Expression of *Eras* in E7.5 neuroectoderm (NE) and primitive streak (PS) detected by real-time RT-PCR (Student's *t*-test; **p < 0.01) (*e*) and western blot (*f*). (*g*) Western blot analysis of ERAS in ESCs cultured in 2i/LIF and LIF/serum medium. (*h*) ERAS protein expression in ESCs cultured in 2i/LIF and 2i/LIF withdrawal for indicated time points. (*i*) Comparison of ERAS protein expression between ESCs cultured in 2i/LIF medium and EpiSCs. (*j*) Real-time RT-PCR analysis of *Eras* mRNA in ESCs cultured in 2i/LIF, N2B27, LIF/serum and EpiSCs (one-way ANOVA; **p < 0.01). (*k*) *Eras* mRNA expression from zygote to blastocyst stage was detected by real-time RT-PCR (one-way ANOVA; **p < 0.01).

In order to screen the functional genes involved in germ layer specification, we performed microarray analysis on E7.5 endoderm, mesoderm and epiblast. Interestingly, we found *Eras* mRNA was highly enriched in endoderm and epiblast compared with its expression in mesoderm of E7.5 embryos (data not shown). To investigate the detailed expression of *Eras* in mouse germ layer specification, we separated the germ layers from E5.5 to E7.5 embryonic regions. Real-time RT-PCR showed that *Eras* mRNA was highly expressed in E5.5–7.5 endoderm and epiblast compared with its expression in E7.5 mesoderm (figure 1*a*). ERAS protein was observed to be dramatically downregulated in mesoderm (figure 1*b*). The location of *Eras* mRNA in E7.5 embryo was further detected by whole-mount *in situ* hybridization. It was enriched in the anterior and extraembryonic regions of E7.5 embryo (figure 1*c*). In the section of the E7.5 embryonic region, *Eras* mRNA concentrated in anterior visceral endoderm and epiblast, in which its expression was higher in neuroectoderm than that in PS (figure 1*d*). When E7.5 epiblast was separated into anterior neuroectoderm and posterior PS by micromanipulation, *Eras* mRNA and protein were observed to be abundantly expressed in the anterior neuroectoderm compared with the PS (figure 1*e,f*).

ESCs are usually cultured in a LIF/serum condition [33], and can also be maintained at ground state in 2i/LIF (CHIR99021, PD0325901 and LIF) medium, which had been used for derivation of ESCs from previously recalcitrant mouse strains and rat [12–14]. Surprisingly, ERAS protein was dramatically decreased in ESCs cultured in 2i/LIF medium compared with the LIF/serum condition (figure 1*g*). In addition, ERAS was increased in the ESCs after 2i/LIF withdrawal from N2B27 medium (figure 1*h*). We also examined the expression of ERAS in epiblast stem cells (EpiSCs). Compared with the expression in ground state ESCs (cultured in 2i/LIF medium), both *Eras* protein and mRNA levels were much higher in EpiSCs (figure 1*i,j*). During mouse pre-implantation development, the expression of *Eras* mRNA was decreased in two-cell embryos, and then significantly increased in blastocysts (figure 1*k*). Because ESCs and EpiSCs are isolated from early stage embryos, they may represent epiblast cells in mouse peri-implantation embryos. Thus, we conclude *Eras* is highly expressed in specific types of cells in mouse peri-implantation embryos.

### 2.2. Establishing ESC lines for *Eras* knockdown and over-expression

**Figure 2.**
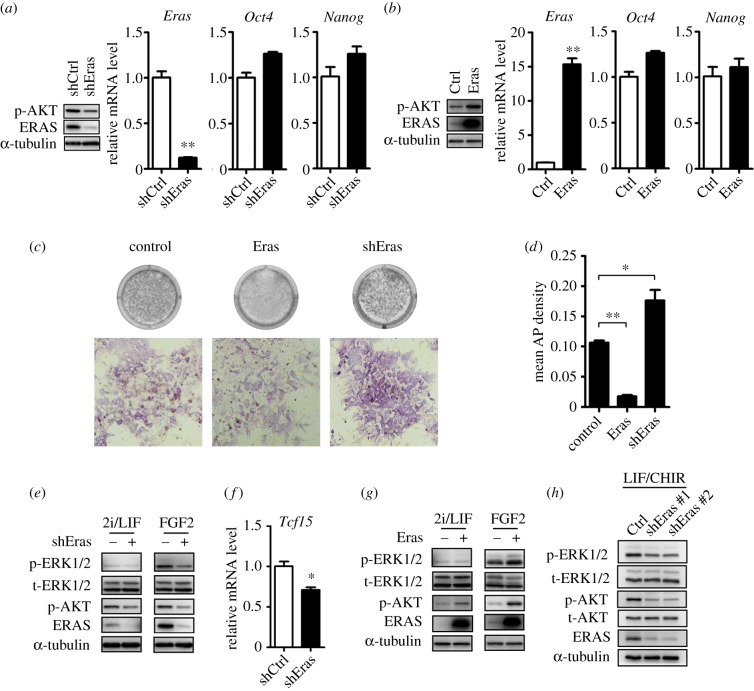
*Eras* was important for ESCs to exit from ground state through regulating ERK activity. (*a,b*) Efficiency of *Eras* knockdown (*a*) and over-expression (*b*) in ESCs cultured in LIF/serum condition. Pluripotency markers showed normal expression (Student's *t*-test; **p < 0.01). (*c,d*) AP staining analysis of ESCs was performed after 3 days of differentiation in N2B27 medium. Weaker AP staining was observed in *Eras* over-expressed ESCs, while *Eras* shRNA ESCs showed increased AP staining, indicating *Eras* expression accelerated the exiting of ESCs from ground state. Mean density was calculated by (IOD SUM)/area (one-way ANOVA; *p < 0.05; **p < 0.01). (*e*) *Eras* knockdown inhibited ERK phosphorylation during ESC commitment. ESCs were cultured in 2i/LIF, or differentiated in N2B27 containing 12 ng ml^−1^ FGF2 for 24 h. (*f*) *Tcf15* expression was decreased during ESC commitment after *Eras* was knocked down. (Student's *t*-test; *p = 0.013). (*g*) *Eras* over-expression increased ERK phosphorylation during ESC commitment. Western blot analysis was performed after ESCs were cultured in 2i/LIF or differentiated in N2B27 containing FGF2 for 24 h. (*h*) Undifferentiated ESCs cultured in LIF/CHIR showed decreased phosphorylation of AKT and ERK in *Eras* shRNA ESCs.

To investigate the function of ERAS, we established mouse ESC lines for the stable knockdown and over-expression of *Eras* by lenti-virus vectors. Compared with the control, the mRNA of *Eras* reduced 88% in the knockdown ESCs and increased approximately 15-fold in the over-expression ESCs (figure 2*a,b*). The efficiencies of knockdown and over-expression of *Eras* were further confirmed by western blot (figure 2*a,b*). *Eras* knockdown and over-expression ESC lines could be routinely passaged in ESC culture medium containing LIF and serum on MEF feeder (data not shown). Consistent with previous findings [26], the *Eras* over-expression cell line grew fast and the knockdown cell line grew slowly (data not shown); knockdown of *Eras* was observed to also inhibit AKT phosphorylation and over-expressed *Eras* accelerated AKT phosphorylation in ESCs (figure 2*a,b*). Real-time RT-PCR results showed that knockdown and over-expression of *Eras* did not significantly affect pluripotent markers expression (figure 2*a,b*). Thus, ESC lines for stable *Eras* knockdown and over-expression were established.

### 2.3. *Eras* is required for efficient ESC commitment through ERK signalling

Beyond its known role in cell growth, the dramatic increase of ERAS in ESCs during the transition from ground into primed state prompted us to investigate its novel function during this transition. To this end, the ground state ESCs were cultured in N2B27 medium on matrigel-coated plates without 2i/LIF to induce differentiation for 3 days. Alkaline phosphatase (AP) staining was performed to assay the undifferentiated ESCs. Compared with the control, significant weaker AP staining was observed in *Eras* over-expression ESCs, suggesting that *Eras* participated in the progression from ground state into primed state (figure 2*c,d*). When *Eras* was knockeddown, more AP positive cells were detected in the differentiating ESCs (figure 2*c,d*). Furthermore, we replated single cells after 3 days of differentiation in 2i/LIF and quantified the percentage of undifferentiated ES cell colonies, which were visualized by AP staining. Compared with control, *Eras* over-expression significantly decreased the clonogenicity of ESCs after a 3-day differentiation, and *Eras* shRNA knockdown cells showed increased colony-forming ability (electronic supplementary material, figure S1). Thus, these data suggest ERAS may also play an important role in the transition of mouse ESCs from ground into primed state.

Ras family proteins function mainly through Raf/MAPK and PI3K/AKT pathways [34,35]. The ERK pathway is one of MAPK pathways, and its inhibition is essential for maintaining the ground state of mouse ESCs [12,15]. To examine if ERAS was involved in the ERK pathway during ESC transition from ground into primed state, we cultured *Eras* knockdown and over-expressed ESCs in N2B27 medium for 24 h by adding FGF2, which had been shown to promote ESC commitment through ERK activation [36], and then analysed phospho-ERK by western blot. *Eras* knockdown inhibited ERK phosphorylation during ESC commitment (figure 2*e*). In addition, *Tcf15*, a critical transcription factor regulated by ERK signalling in the primed state [37], was significantly decreased by *Eras* knockdown during the commitment of the ESCs for 24 h (figure 2*f*). Furthermore, *Eras* over-expression slightly enhanced phospho-ERK level during the commitment of ESCs (figure 2*g*). To detect the role of *Eras* on ERK phosphorylation in undifferentiated ESCs, we cultured *Eras* knockdown ESCs in LIF/CHIR condition. Compared with the control, phospho-ERK was also decreased in *Eras* knockdown ESCs (figure 2*h*). Thus, these results show that *Eras* regulates ERK phosphorylation in both undifferentiated ESCs and differentiating ESCs from ground state.

ERAS has been shown to directly interact with PI3K (p110*δ*), but not bind to Raf, to activate the AKT pathway [26]. To examine if ERAS regulated ERK activity through the AKT pathway, Ly294002, a highly specific inhibitor of PI3K, was added to the culture medium. Ly294002 could efficiently inhibit AKT phosphorylation, but did not affect ERK activity (electronic supplementary material, figure S2). Thus, ERK is not a direct target of AKT during ESC commitment from ground state into primed state.

### 2.4. Downregulated *Eras* is important for primitive streak and mesoderm formation

Considering the specific low expression of *Eras* in PS and mesoderm (figure 1*a–f*), we assumed that the downregulated expression of this gene also may be important for the specification of PS and mesoderm. Compared with ground ESCs, ESCs cultured in LIF/serum medium have distinct transcriptome and epigenome profiles [38]. These cells represent a relatively primed and metastable state without ERK inhibition, and recapitulate the overall methylation pattern of epiblast cells [39–41]. In our experiments, we also observed increased epiblast markers (*Fgf5* and *Tcf15*) expression in ESCs cultured in LIF/serum (electronic supplementary material, figure S3*a*). Furthermore, we performed spontaneous differentiation of control and *Eras* knockdown ESCs in serum containing medium, starting from ESCs cultured in 2i/LIF and LIF/serum. Primitive steak marker (*T*) expression showed more rapid upregulation in *Eras* knockdown ESCs differentiated from the LIF/serum condition (electronic supplementary material, figure S3*b,c*), supporting that ESCs cultured in LIF/serum represent a relatively primed state [39]. Considering that *Eras* functions in the ESC commitment from ground into primed state (figure 2), it might not be appropriate to use ground state ESCs for *Eras* functional analysis during germ layer specification. Therefore, we used ESCs cultured in LIF/serum medium as an original condition to investigate the role of *Eras* on germ layer specification during embryoid body (EB) formation and cytokine-induced differentiation.

**Figure 3.**
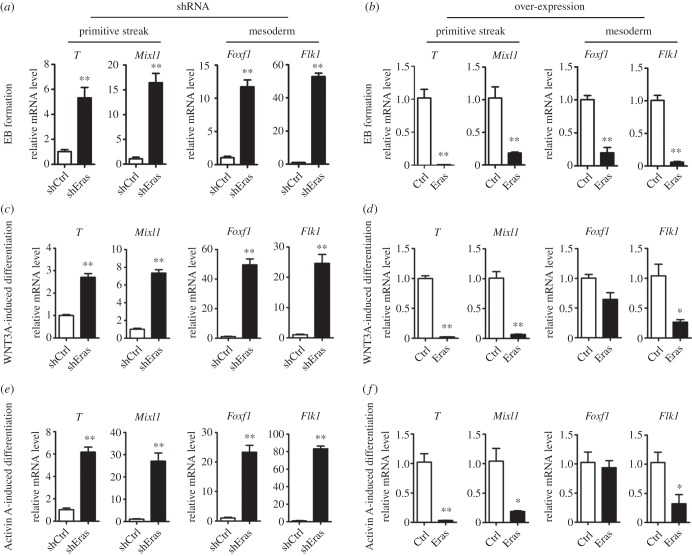
*Eras* negatively regulated the differentiation of ESCs into primitive streak and mesoderm. (*a*) *Eras* knockdown promoted the differentiation of ESCs into primitive streak and mesoderm during EB formation. (*b*) *Eras* over-expression inhibited the differentiation of ESCs into primitive streak and mesoderm during EB formation. (*c–f*) *Eras* negatively regulated the differentiation of ESCs into primitive streak and mesoderm induced by WNT3A and Activin A, respectively. Primitive streak markers: *T* and *Mixl1*; mesoderm markers: *Flk1* and *Foxf1* (Student's *t*-test; **p < 0.01; *p < 0.05).

Although there were some differences in the cell growth, *Eras* over-expression and knockdown ESCs formed grossly normal EBs (data not shown). Compared with the control, *Eras* knockdown dramatically increased the expression of markers in of PS (*T* and *Mixl1*), mesoderm (*Flk1* and *Foxf1*) and endoderm (*Sox17* and *Foxa*2), and decreased neuroectoderm markers (*Sox1* and *Pax6*) expression during EB formation (figure 3*a*; electronic supplementary material, figure S4*a*). In addition, over-expressed *Eras* significantly decreased the expression of mesoderm and PS markers in this ESC differentiation system (figure 3*b*), but the neuroectoderm and endoderm markers were not affected significantly (electronic supplementary material, figure S4*b*). These results suggest that downregulated expression of *Eras* facilitates the differentiation of PS and mesoderm cells.

To further confirm the role of ERAS on the formation of PS and mesoderm, we employed a cytokine-induced differentiation system [42]. WNT3A and Activin A were used to induce ESC differentiation into PS and mesoderm lineages [43]. In these systems, the expression of PS and mesoderm markers was significantly increased during cytokine-induced differentiation in *Eras* knowndown ESCs. In contrast, the expression of PS and mesoderm markers was significantly decreased in the ESCs with over-expressed *Eras* during the differentiation (figure 3*c–f*).

**Figure 4.**
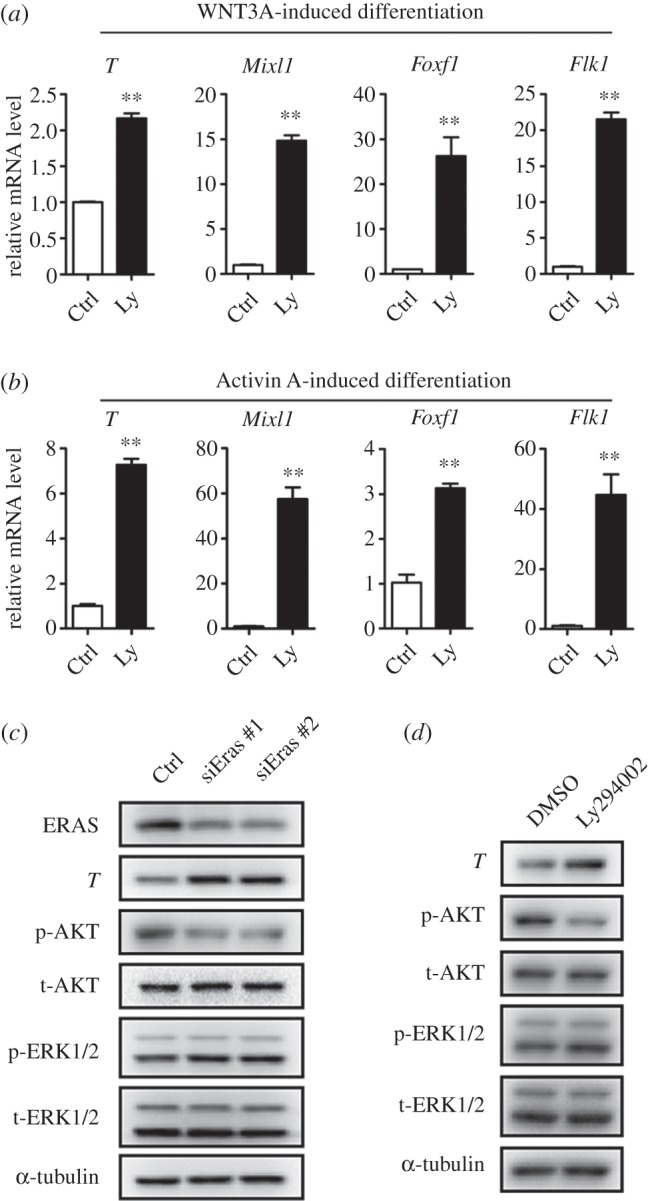
*Eras* inhibited the differentiation of ESCs and EpiSCs into primitive streak and mesoderm through AKT activation. (*a,b*) Inhibition of AKT by Ly294002 (Ly, 10 µM) promoted WNT3A or Activin A induced primitive streak and mesoderm differentiation (Student's *t*-test; **p < 0.01). (*c*) *Eras* knockdown facilitated primitive streak differentiation in EpiSCs and inhibited AKT activity. (*d*) Inhibition of AKT by Ly294002 (10 µM) promoted primitive streak marker *T* expression in EpiSCs.

Although the inhibition of PI3K did not affect the activity of ERK in ESCs (electronic supplementary material, figure S2), the inhibition of PI3K and AKT was reported to promote the differentiation of human pluripotent stem cells [44–46]. We then examined the role of Ly294002 on cytokine-induced differentiation of mouse ESCs. Ly294002 efficiently promoted the specification of PS and mesoderm after WNT3A or Activin A treatment (figure 4*a,b*). Furthermore, we found that *Eras* knockdown also decreased AKT phosphorylation and promoted PS marker (*T*) expression in mouse EpiSCs, without effect on ERK activity (figure 4*c*). Consistently, Ly294002 treatment decreased AKT phosphorylation and increased *T* expression in mouse EpiSCs (figure 4*d*). Thus, these results suggest that *Eras* functions through AKT during the formation of PS and mesoderm.

Altogether, these results with different ESC differentiation systems consistently support that *Eras* downregulation is important for the formation of PS and mesoderm, suggesting the role of downregulated *Eras* during mouse early embryonic development.

### 2.5. *Eras* is negatively regulated by β-catenin in stem cells

**Figure 5.**
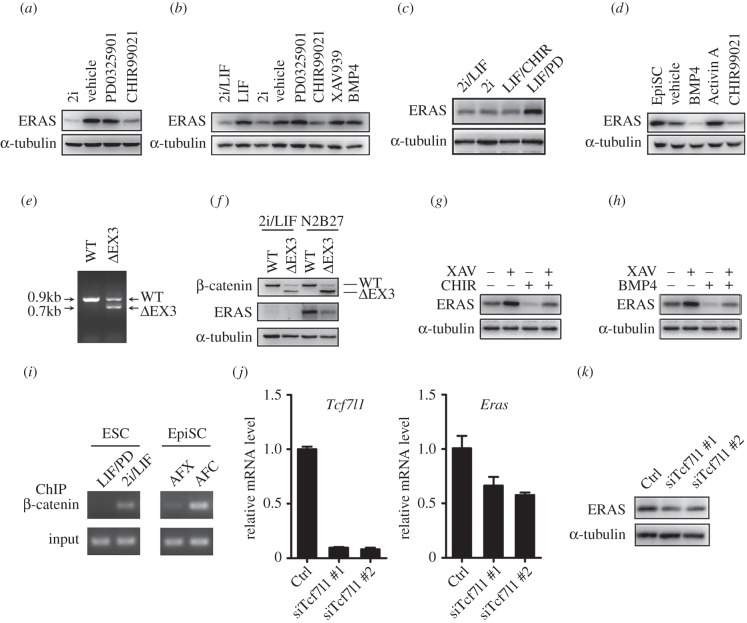
ERAS expression was negatively regulated by β-catenin in ESCs and EpiSCs. (*a*) ERAS expression was inhibited by CHIR99021 in ESCs cultured in N2B27 medium. ESCs were treated with the inhibitors for 24 h. (*b*) ERAS expression in ESCs was inhibited by CHIR99021 treatment in DMEM/serum medium. ESCs were treated with indicated cytokines or inhibitors for 24 h. (*c*) ERAS expression was decreased in the presence of CHIR99021 in undifferentiated ESCs by mixing of two factors. (*d*) ERAS expression in EpiSCs was inhibited by CHIR99021 and BMP4 treatment for 24 h. (*e*) Genotyping of wild-type and *Catnb*^(*ΔEx3/+*)^ ES cell lines. β-catenin with exon 3 deletion created a constitutive activated form of β-catenin. Wild-type = 0.9 kb, ΔEx3 = 0.7 kb. (*f*) ERAS protein was decreased in *Catnb*^(*ΔEx3/+*)^ ESCs cultured in N2B27 for 24 h. (*g*) The inhibitory effect of CHIR99021 on ERAS expression was attenuated by XAV939 treatment in EpiSCs. (*h*) The inhibitory effect of BMP4 on ERAS expression was partially counteracted by XAV939 treatment in EpiSCs. (*i*) β-Catenin was recruited to the *Eras* promoter after GSK3 inhibition. ESCs were cultured in LIF/PD or 2i/LIF. EpiSCs were cultured in AFX (Activin A, FGF2 and XAV939) or AFC (Activin A, FGF2 and CHIR99021). (*j,k*) After *Tcf7l1* knockdown for 24 h, *Eras* mRNA and protein were decreased in EpiSCs. Cytokines and inhibitors were used at the indicated final concentrations: PD0325901 (1 µM), CHIR99021 (3 µM), XAV939 (2 µM), LIF (1000 units ml^−1^), BMP4 (10 ng ml^−1^) and Activin A (20 ng ml^−1^).

The dynamic expression pattern of *Eras* during embryonic development and the important roles of this gene during cell growth and specification prompted us to investigate the regulation of ERAS expression. *Eras* was inhibited in 2i medium (CHIR99021 and PD0325901) (figure 1*g,h*), indicating these two inhibitors regulated the expression of *Eras*. To investigate this possibility, we cultured the ESCs in N2B27 with 2i, CHIR99021 or PD0325901. We found ERAS was inhibited by CHIR99021, but not PD0325901, in N2B27 medium (figure 5*a*). Furthermore, in the ESCs cultured in DMEM/serum medium, CHIR99021, but not other factors, was found to significantly inhibit ERAS expression (figure 5*b*). In order to exclude the indirect effect of differentiation on ERAS expression, we detected ERAS expression in the undifferentiated ESCs by mixing two of three factors (PD0325901, CHIR99021 and LIF). ERAS was also decreased in the presence of CHIR99021 (figure 5*c*), indicating the effect of CHIR99021 on ERAS expression was not dependent on the differentiation state. However, in EpiSCs, not only CHIR99021 but also BMP4 could dramatically inhibit ERAS expression (figure 5*d*). Thus, CHIR99021 is a major factor for the downregulation of *Eras* in both ESCs and EpiSCs.

CHIR99021, a specific inhibitor for GSK3, functions in cells by regulating metabolic activity and controlling β-catenin transcription factor activity [12]. To investigate how CHIR99021 regulates the expression of *Eras*, we established a *Catnb*^(*ΔEX3/+*)^ ESC line (figure 5*e,f*), which expressed active β-catenin constitutively [47]. Consistent with our previous findings (figure 1*g,h*), ERAS showed low expression in wild-type and *Catnb*^(*ΔEX3/+*)^ ESCs cultured in 2i/LIF medium (figure 5*f*). However, compared with the control, ERAS expression was dramatically decreased in *Catnb*^(*ΔEX3/+*)^ ESCs cultured in N2B27 medium (figure 5*f*). These results indicate active β-catenin inhibits ERAS expression in ESCs.

*Catnb*^(*ΔEX3/+*)^ EpiSCs could not be established because of an early embryonic lethal effect (data not shown). XAV939 is a tankyrase (TNKS) inhibitor which retains β-catenin in the cytoplasm to antagonize WNT signalling in EpiSCs [48]. To further investigate whether CHIR99021 inhibits *Eras* expression through β-catenin in EpiSCs, we cultured EpiSCs in CHIR/XAV conditions and examined the expression of ERAS. XAV939 increased the expression of ERAS and restored the ERAS expression inhibited by CHIR99021 (figure 5*g*). These results indicated β-catenin regulates the expression of *Eras* in EpiSCs.

In EpiSCs, ERAS was also downregulated by BMP4 (figure 5*c*). Consistent with this observation, BMP4 dramatically inhibited *Eras* expression in wild-type EpiLCs (epiblast-like cells), but not in *Wnt3* knockout cells during germ cell differentiation *in vitro* [49] (electronic supplementary material, figure S5), indicating BMP4 may regulate *Eras* expression through the Wnt/β-catenin pathway in EpiSCs. To this end, we treated EpiSCs with BMP4 and XAV939. We observed that XAV939 partially counteracted the inhibition by BMP4 of ERAS expression in EpiSCs (figure 5*h*). These results indicate BMP4 inhibits *Eras* expression through the β-catenin pathway in EpiSCs, consistent with a previous report that β-catenin signalling was induced by BMP4 in EpiSCs [50].

In order to confirm the transcriptional regulation of *Eras* by β-catenin, chromatin immunoprecipitation was used to detect the interaction between β-catenin and *Eras* promoter. ESCs were cultured in LIF/PD or 2i/LIF (GSK3 inhibition). EpiSCs were cultured in AFX (Activin A, FGF2 and XAV939) or AFC (Activin A, FGF2 and CHIR99021; GSK3 inhibition). β-Catenin was recruited to the promoter of *Eras* in both ESCs and EpiSCs after GSK3 inhibition by CHIR99021 (figure 5*i*). Inhibition of GSK3 supports mouse ESC self-renewal by modulating β-catenin*–*TCF3 interaction (*Tcf7l1* was known as *Tcf3* previously) [51–53]. After knocking down *Tcf7l1* in ESCs, *Eras* mRNA and protein was decreased (figure 5*j,k*), indicating *Tcf7l1* is involved in *Eras* expression.

In conclusion, these results suggest the expression of *Eras* is negatively regulated by β-catenin in stem cells.

### 2.6. *Eras* is downregulated by β-catenin in early mouse embryos

**Figure 6.**
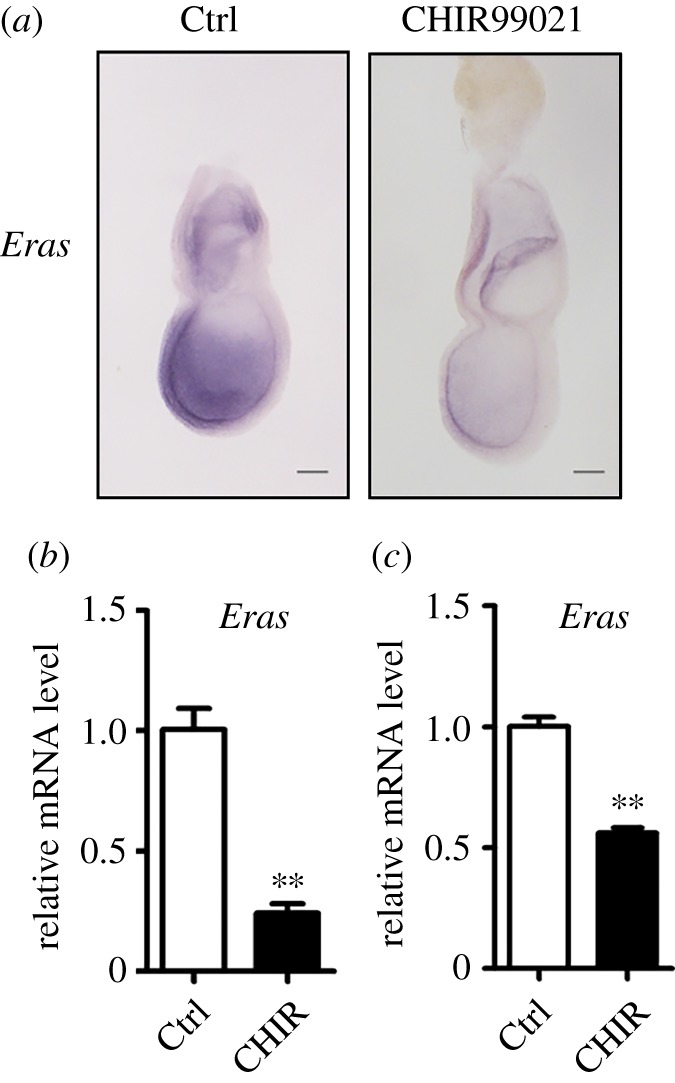
*Eras* expression in embryos was inhibited by CHIR99021 treatment. (*a*) *In situ* hybridization showed that *Eras* mRNA was decreased in E6.5 embryos treated with CHIR99021 (30 µM) for 15 h. Scale bar, 100 µm. (*b*) *Eras* mRNA was detected with real-time RT-PCR in E6.5 embryos treated with CHIR99021 (30 µM) for 15 h. (*c*) CHIR99021 (3 µM) treatment for 24 h decreased *Eras* expression in blastocysts cultured *in vitro*. (Student's *t*-test; **p < 0.01).

To explore the role of β-catenin on *Eras* expression physically, we cultured E6.5 embryos in medium containing CHIR99021 for 15 h and examined the *Eras* expression by whole-mount *in situ* hybridization and real-time RT-PCR. It was reported that *Sox2* expression was inhibited by CHIR99021 in embryonic ectoderm but not extraembryonic ectoderm [54]. However, upon the treatment with CHIR99021, *Eras* expression in the embryos was decreased in both embryonic and extraembryonic ectoderm (figure 6*a*). Combining with the inhibitory effect of CHIR99021 on *Eras* also in both ESCs and EpiSCs, we conclude the regulation of *Eras* expression in these cells may be conserved. Real-time RT-PCR results also showed that *Eras* expression was significantly decreased in the embryos after the treatment with CHIR99021 (figure 6*b*). To examine if β-catenin regulates *Eras* expression in pre-implantation embryos, blastocysts recovered from normal mice at E3.5 were treated with CHIR99021 for 24 h. Compared with the control, *Eras* mRNA was significantly decreased in the blasocysts treated with CHIR99021 (figure 6*c*). In accordance with the downregulation of *Eras* expression by CHIR99021 and the low expression of *Eras* in PS and mesoderm, β-catenin was observed to be specifically activated in these cell lineages [55]. These results indicate that the activated β-catenin downregulates the expression of *Eras* in mouse early embryos.

## 3. Discussion

After mouse embryo implantation, cell number was dramatically increased accompanying cell differentiation [56], suggesting some specific regulators function during this process. Although mouse transgenesis tremendously advances the molecular insights into mammalian early embryonic development, the researches on numerous genes whose mutations in mice do not result in obvious phenotypes are hindered by these analyses [57]. *Eras* was initially screened as a Ras family protein specifically expressed in ESCs, and its mutation does not lead to an obvious phenotype in mice [26]. However, *Eras* does play an important role in ESC growth [26] and promotes somatic cell reprogramming [27], suggesting its possible roles in mouse early embryonic development [28]. In this study, we find that *Eras* is highly expressed in early mouse peri-implantation embryos, while it is specifically downregulated in PS and mesoderm of E7.5 embryos. Beyond its role on cell growth, upregulated *Eras* facilitates the differentiation of ESCs from ground to primed state, and also downregulated *Eras* expression is important for the formation of PS and mesoderm. In addition, we find the expression of *Eras* is tightly regulated by the β-catenin pathway in ESCs and mouse early embryos. Thus, these results indicate that *Eras* probably coordinates cell proliferation and cell differentiation during mouse early embryonic development.

The ERK pathway plays a critical role during the commitment of ground state ESCs into the primed state. The inhibition of this pathway maintains the ground state of ESCs, and the activation of ERK signalling triggers the transition of ESCs from self-renewal to lineage commitment [12,15,17,58]. However, how the ERK pathway is regulated during the ESC commitment from ground into primed state is still largely unclear. Interestingly, we find that over-expressed *Eras* stimulates ERK phosphorylation and knockdown of *Eras* significantly decreases the phosphorylation of ERK during ESC commitment from ground into primed state, suggesting that ERAS is an important regulator that is involved in the ERK pathway in this process. Currently, ERAS is only known to bind to PI3K (p110*δ*) and increase AKT phosphorylation to promote ESC growth and the formation of iPSCs [26,27]. In accordance with these observations, we find that over-expressed *Eras* stimulates the phosphorylation of AKT and *Eras* knockdown decreases AKT phosphorylation. However, Ly294002, a specific inhibitor of PI3K, significantly inhibits phosphorylation of AKT but not ERK phosphorylation in ESCs. Thus, these results indicate that *Eras* probably regulates ERK activity through an unknown mechanism.

AKT inhibitor has been widely used for mesendoderm induction in human embryonic stem cells [44–46], but developmental evidence for using this inhibitor is still lacking. In this study, we find over-expressed *Eras* dramatically increases AKT phosphorylation and inhibits the formation of PS and mesoderm. In addition, knockdown *Eras* significantly decreases AKT activity and promotes the formation of PS and mesoderm. It is well known that β-catenin regulates the formation of mesoderm during mouse early embryonic development [59,60]. Considering that over-expression of *Eras* inhibited the differentiation of PS and mesoderm induced by WNT3A (figure 3*d*), ERAS may link the β-catenin and AKT pathways in the formation of PS during mouse early embryonic development. As described above, ERAS regulates both AKT and ERK activities in ESCs, while regulating only AKT activity and not ERK activity at later development stages. How ERAS achieves the specificity during these processes deserves to be further investigated.

Over-expressed *Eras* has been shown to increase tumour formation in NIH 3T3 cells [26]. Increased *ERAS* expression is also observed in human gastric carcinoma and neuroblastoma cells, and its expression is significantly associated with metastasis to the lymph nodes and liver. Neuroblastoma cells transfected with *ERAS* expression vector showed resistance to chemotherapeutic agents and promotion of transforming activity [29–32]. Thus, ERAS may be an important regulator for some types of human cancer. We find that CHIR99021 and BMP4 significantly decrease the expression of *Eras*, suggesting these pathways may be candidates for the treatment of some types of human cancer.

The emerging iPSC (induced pluripotent stem cell) technology has solved the ethical issues, may overcome the immunogenicity of cell therapy and promotes the application of stem cells in clinics [61,62]. Directed differentiation of ESCs/iPSCs into cells safe for clinical use is the next challenge in stem cell research. Manipulating the key pathways that regulate the formation of germ layers of mouse embryos resulted in the induction of endoderm, mesoderm and ectoderm cells from ESCs [43,63]. Recently, pancreatic β cells have been successfully generated from human pluripotent stem cells by a specific process during which the first step is the generation of definitive endoderm by manipulating *Wnt* and *Nodal* pathways [64]. Thus, understanding the molecular mechanisms of germ layer specification during early embryonic development will significantly accelerate the strategy of directing ESC differentiation into specific cell lineages *in vitro* [65]. Our findings described here show the dynamic expression of ERAS during the cell specification of mouse early embryos and ESC differentiation. The precise regulation of ERAS expression is important for ESC growth and commitment from ground to primed state, and PS and mesoderm formation. Thus, also, ERAS may be an important target for the differentiation of ESCs into specific lineage cells.

Although ERAS knockdown affects ESC growth and differentiation, no overt developmental phenotype was observed in *Eras*-deficient mice [26]. The plasticity of mouse early embryonic development and the redundancy of Ras family genes may account for this [28]. It should also be noted that numerous genes involved in ESC function are not essential for mouse early development. For example, the bHLH transcription factor *Tfe3* mutant mice are viable and fertile [66], while later research showed that *Tfe3* is very important for the exiting of ESCs from the ground state [67].

In conclusion, our results illustrate that *Eras* is important for early cell lineage specification and provide new molecular insights into mouse early embryonic development and how *Eras* may contribute to ESC differentiation.

## 4. Material and methods

### 4.1. Antibodies and reagents

Antibodies against phospho (Ser473)-AKT1 (#9018), AKT1 (#2938), phospho (Thr202/Tyr204)-ERK1/2 (#4370), ERK1/2 (#4695), phospho (Ser465/467)-SMAD2 (#3108), SMAD2 (#5339) and *α*-tubulin (#2144) were purchased from Cell Signaling Technology. Anti-ERAS (sc-51072) was purchased from Santa Cruz Biotechnology. Anti-β-catenin antibody (ab6302) was from Abcam Company. Inhibitors and cytokines include Ly294002 (#9901, CST), PD0325901 (P-9688, LC Laboratories), CHIR99021 (C-6556, LC Laboratories), XAV939 (X3004, Sigma), LIF (ESG1107, Millipore), BMP4 (5020-BP-010/CF, R&D Systems), Activin A (120-14, Peprotech) and FGF2 (233-FB-025, R&D Systems).

### 4.2. Mice maintaining and germ layer separation

CD1 mice were housed under specific-pathogen-free (SPF) conditions (12 L : 12 D cycles) in the animal facilities at the Institute of Zoology, Chinese Academy of Sciences. They were raised with standard laboratory food and water.

Embryos from E5.5 to E7.5 were dissected from the deciduas in DMEM buffered with 25 mM HEPES (pH 7.4) containing 10% FBS. Reichert's membrane was removed with fine forceps. The embryonic region was cut off using a glass scalpel. The embryonic region was rinsed in Dulbecco's phosphate-buffered saline (DPBS), and incubated in pancreatic/trypsin enzyme solution (a solution of 0.5% trypsin and 2.5% pancreatin in Ca^2+^/Mg^2+^-free Tyrode Ringer's saline, pH 7.6–7.7) at 4°C for 8–12 min. After the treatment, the embryonic region was transferred into DMEM buffered with 25 mM HEPES (pH 7.4) containing 10% FBS. The germ layers were separated carefully with Pasteur pipette and glass needles as previously reported [68,69].

### 4.3. RNA isolation and real-time RT-PCR

Total RNA was isolated by an RNeasy mini kit (Qiagen). Concentration of RNA was measured using a NanoDrop 2000 (Thermo Fisher). The reverse transcription reaction was performed using PrimeScript RT Reagent Kit (RR037A, TaKaRa). Real-time PCR was performed using a SYBR Premix Ex Taq kit (DRR041S, TaKaRa), and *Hprt* was used as an internal reference. The 2^−ΔΔCt^ method was used for data analysis [70]. Primer sequences for real-time RT-PCR are listed in the electronic supplementary material, table S1.

### 4.4. Whole-mount *in situ* hybridization

E7.5 embryos from CD1 pregnant mice were dissected from the deciduas, and Reichert's membrane was removed. The embryos were fixed in a clean vial containing fresh 4% paraformaldehyde in PBS at 4°C overnight and dehydrated through methanol series. Antisense probes for *Eras* were labelled with DIG RNA Labeling Kit (Roche). The procedure of whole-mount *in situ* hybridization and sectioning was performed as previously reported [71].

### 4.5. Western blot

Cells were rinsed in PBS, and then lysed with RIPA lysis buffer containing complete protease inhibitor cocktail (Roche) and phosphatase inhibitors (NaF and Na_3_VO_4_). Sample viscosity was reduced by sonication for 10 s to shear DNA. BCA reagent kit (Beyotime) was used to measure protein concentration. Normalized samples were run on a 12% PAGE gel and transferred onto PVDF membranes. After blocking for 1 h in 5% non-fat milk, the membranes were incubated with primary antibodies (1 : 1000) at 4°C overnight. After three washes in TBST, the membranes were incubated with secondary antibodies conjugated with horseradish peroxidase at room temperature for 1 h. The signals were developed with Pierce ECL Western Blot Substrate in Bio-RAD Chemi DocTMXR^s^+ (Bio-Rad Laboratories, USA) with Quantity One software.

### 4.6. Mouse embryonic stem cells culture and differentiation

Mouse embryonic stem cell line (CMTI-1, Millipore) was routinely cultured in the dishes coated with MEF (mouse embryonic fibroblasts) feeder treated with mitomycin C under 5% CO_2_ at 37°C. The ESC culture medium included DMEM supplemented with 15% ESC-qualified FBS (Millipore), 2 mM l-glutamine, 100 units ml^−1^ penicillin/streptomycin, 0.1 mM non-essential amino acids, 0.1 mM 2-mercaptoethanol, 1 mM sodium pyruvate and 1000 units ml^−1^ LIF (Esgro, Cat# ESG1107, Millipore). The ground state ESCs were cultured in gelatin-coated dishes without feeder with 2i/LIF medium, which included N2B27 medium supplemented with PD0325901 (1 µM), CHIR99021 (3 µM) and LIF (1000 units ml^−1^) [12].

The EB formation was performed in a hanging drop culture system [72]. Briefly, ESCs were dissociated with trypsin-EDTA (0.25%) and replated to remove the feeders. After an initial culture for 1 h, ESCs were collected and diluted to 2.0 × 10^4^ cells ml^−1^ in standard ESC culture medium lacking LIF. Then the ESCs were allowed to aggregate in hanging droplets (30 µl) and cultured for 3 days before being harvested for mRNA analysis.

Cytokine-induced differentiations were performed as previous reports [42]. After removing MEF feeder, to start monolayer differentiation, ESCs were plated at a density of 1 × 10^4^ cells cm^−2^ in N2B27 medium overnight on gelatin-coated culture dishes to form a monolayer, and then were treated with Activin A (20 ng ml^−1^) and WNT3A (10 ng ml^−1^) respectively to induce differentiation for 3 days. The differentiated cells were collected for RNA and protein analyses. The medium with Activin A or WNT3A was changed every day until RNA and protein isolation.

### 4.7. Establishing epiblast stem cell line and *Catnb*^(*ΔEX3/+*)^ ES cell line

Mouse E5.75 embryos (129S2 × C57BL/6, F1) were dissected from uteri and epiblasts were obtained through enzyme digestion and micromanipulation as previously described [69,73]. The epiblasts were cultured on MEF feeder in N2B27 medium containing Activin A (20 ng ml^−1^, Peprotech), bFGF (FGF2, 12 ng ml^−1^, R&D), XAV939 (2 µM, Sigma-Aldrich) and 20% KSR (Gibco) [54,74]. After 2–3 days of culture, the epiblast outgrowths were ready for passage. For routine passage, EpiSCs were split 1 : 4–1 : 6 every 2–3 days by dissociating with collagenase IV (1 mg ml^−1^, Gibco) as cell clumps.

To verify the function the *Eras* in EpiSCs, the cells were plated in matrigel-coated dishes for *Eras* knockdown. Transfection was performed with lipofectamine 2000 (Invitrogen) according to the manufacturer's instruction. *Eras* siRNAs were ordered from Invitrogen according to the sequences (#1, 5′ GAGGCTACATTCTAAATGT 3′; #2, 5′ GCTTCGTGAAAGACCATGA 3′). After transfection for 6 h, the medium was changed with N2B27 containing 2 ng ml^−1^ Activin A for PS induction. Protein samples were collected for western blot analysis after 24 h differentiation. For *Tcf7l1* knockdown, *Tcf7l1* siRNA sequences were synthesized according to the following sequences (#1, 5′ GCACCTACCTACAGATGAA 3′; #2, 5′ GCCTTCATGTTGTATATGA 3′).

For *Catnb^(ΔEX3/+)^* ESC line establishment, C57BL/6 wild-type female mice were mated with male *Catnb^lox(ex3)^·Prm-Cre* mice (specific knockout in the male germ line), and blastocysts were cultured in 2i/LIF medium [12]. After a 5-day culture, the colonies were dissociated with TrypLE (Gibco) and transferred to a 96-well plate with MEF feeder. Routine passage was performed for clonal expansion of the ESCs. Genotype was confirmed by PCR with specific primers (GF2: 5′ GGACAATGGCTACTCAAGGTTTGTG 3′ and AS: 5′ ACGTGTGGCAAGTTCCGCGTCATCC 3′) and western blot with rabbit anti-β-catenin antibody (ab6302).

### 4.8. Establishing knockdown and over-expressed *Eras* ES cell lines

For *Eras* knockdown, *Eras* RNAi sequences (#1, 5′ GAGGCTACATTCTAAATGT 3′; #2, 5′ GCTTCGTGAAAGACCATGA 3′) were cloned into psicoR lenti-virus vector, respectively. *Eras* CDS region was cloned into pHIV-EGFP (Addgene) lenti-virus vector for *Eras* over-expression. Lenti-virus was produced by co-transfection of expression vector, envelop plasmid pMD2G and packaging plasmid psPAX2 in 293-FT cells and concentrated by centrifugation at 20 000 rpm for 2 h. EGFP positive ESC clones were screened with flow cytometry for stable expression. The expression and RNAi efficiency was determined by real-time RT-PCR with primers (electronic supplementary material, table S1) for *Eras* and western blot with rabbit anti-ERAS (sc-51072).

### 4.9. ES cell differentiation from ground state and AP staining

ESCs were plated in 2i/LIF medium on matrigel-coated plates at 1.5 × 10^4^ cells cm^−2^. The next day, cells were washed with DPBS to remove the residual inhibitors and LIF, and the medium was changed to N2B27 without 2i/LIF. After 3 days of differentiation, the undifferentiated ESCs were assayed by AP staining. For AP staining, cells were fixed in 4% paraformaldehyde and then incubated with BCIP/NBT for 15 min in AP1 solution (0.1 M NaCl, 0.1 M Tris pH 9.5, 50 mM MgCl_2_, Tween 0.1%). Integrated optical density (IOD) and area were analysed with IMAGE-PRO PLUS software. Mean density was calculated by (IOD SUM)/area.

### 4.10. Chromatin immunoprecipitation

ESCs and EpiSCs were cross-linked with formaldehyde. The chromatin was sheared by sonication with eight rounds of 15 1-s pulses at 60% power output [75]. The following procedure was performed as previously reported [76]. Anti-non-phospho (active) β-catenin (1 : 100, #8814, CST) was used for chromatin immunoprecipitation. RT-PCR was performed for semi-quantitative analysis. Primers for *Eras* promoter (−2395 to −2277) amplification are as follows: Eras-ChIP-F: AACAGAAGAGGGGTATTGGGTAG; Eras-ChIP-R: TGGGCATTGACATAGCGACT.

### 4.11. Mouse embryo culture

E6.5 Embryos from CD1 pregnant mice were dissected from the deciduas. After Reichert's membrane was removed, the embryos were cultured in CMRL 1066 (Gibco) containing 50% rat serum (derived from dorsal aorta) in the presence or absence of 30 µM CHIR99021. After 15 h culture, the embryos were collected for whole-mount *in situ* hybridization and real-time RT-PCR analysis. E3.5 blastocysts were flushed from uteri, cultured in N2B27 containing 3 µM CHIR99021 for 24 h and were examined by real-time RT-PCR with specific primers for *Eras*.

### 4.12. Statistical analyses

Quantitative analyses were based on at least three independent biological samples and expressed as the mean ± s.e.m. Statistics were calculated using SPSS 18.0. The data were subjected to Student's *t*-test or one-way analysis of variance (ANOVA), with a significance level of *p* < 0.05.

## Supplementary Material

Supplementary Material-ERAS regulates mouse development

## Data accessibility

Relevant data supporting this article have been uploaded as part of the electronic supplementary material.
