# Supplementary material for: The roles of ERAS during cell lineage specification of mouse early embryonic development
Source: Open Biol. 2015 Aug 12;5(8):150092. doi: 10.1098/rsob.150092 (PMC4554925; doi:10.1098/rsob.150092)
Supplement: Supplementary Material-ERAS regulates mouse development [file rsob150092supp1.pdf]

Supplementary Material

Supplementary Figures

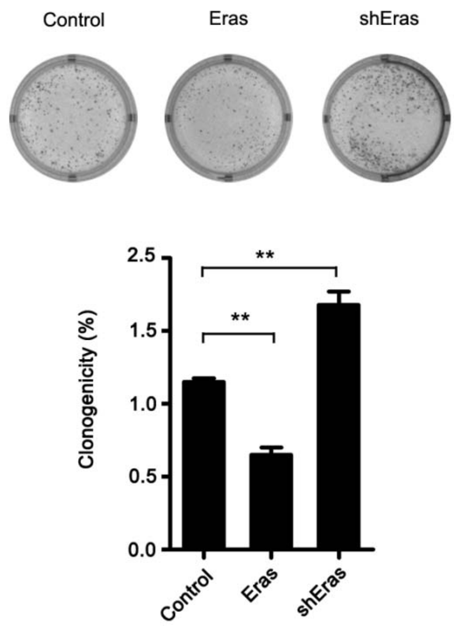

Figure S1. ES cell lines (Control, *Eras* over-expression and *Eras* shRNA knockdown) differentiated for 72 hours in N2B27 were replated in 2i/LIF for 3 days. ESC colonies were visualized by AP staining. Average clonogenicity and s.e.m. were relative to number of plated cells. (One way ANOVA; **, p<0.01).

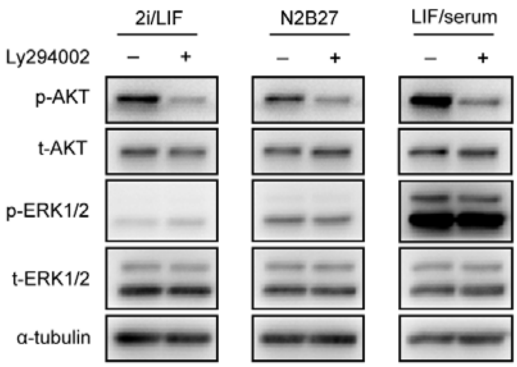

Figure S2. Ly294002 (10 μM) inhibited AKT phosphorylation after 6 hours treatment, but ERK activity was not affected in ESCs cultured in 2i/LIF, N2B27 and LIF/serum, respectively.

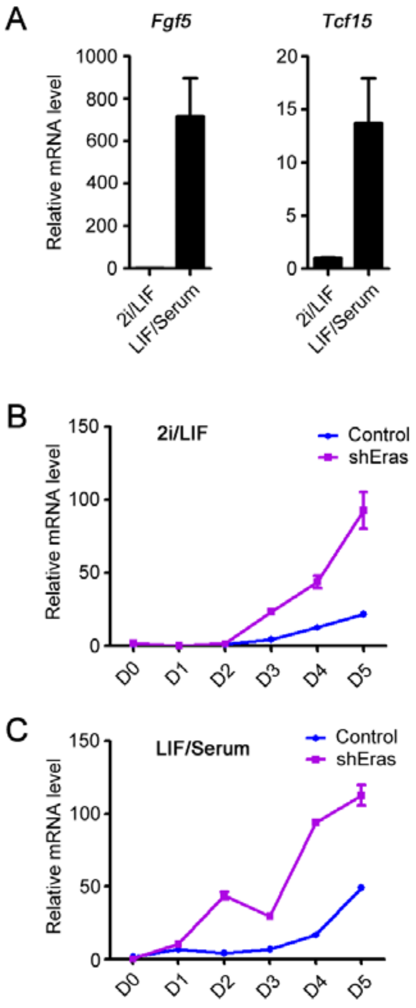

Figure S3. Difference between ground state ESCs and ESCs cultured in LIF/serum. (A) Epiblast markers (*Fgf5* and *Tcf15*) were highly expressed in ESCs cultured in LIF/serum. (B) ESCs (control and shEras) starting from 2i/LIF differentiated spontaneously in serum containing medium for indicated time points. Primitive steak marker (*T*) expression was examined by real-time RT-PCR. (C) ESCs (control and shEras) starting from LIF/serum differentiated spontaneously in serum containing medium for indicated time points. Relative *T* mRNA expression was examined.

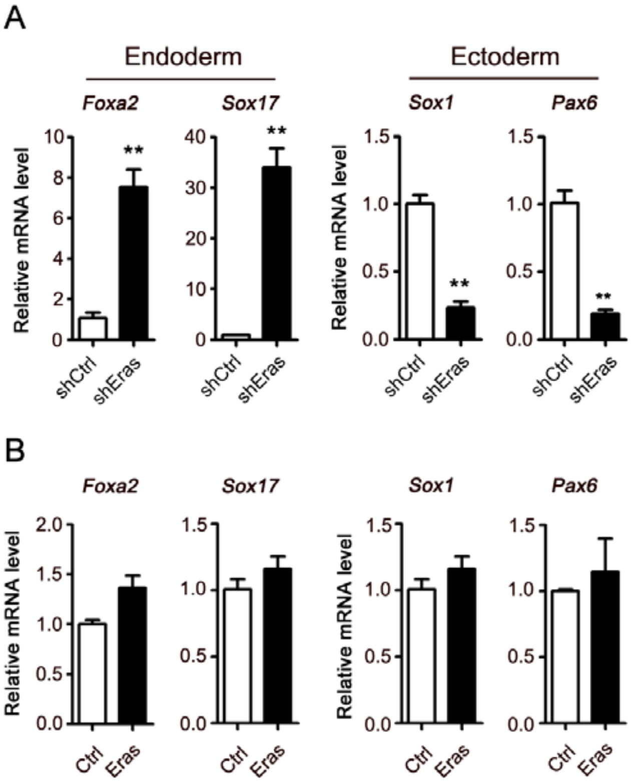

Figure S4. Effects of *Eras* on endoderm and ectoderm differentiation during EB formation. (A) *Eras* knockdown EB showed increased endoderm markers and decreased ectoderm markers expression. (B) *Eras* over-expression had no effect on endoderm and ectoderm markers expression. Endoderm markers: *Sox17* and *Foxa2*; Neuroectoderm markers: *Sox1* and *Pax6* (Student's t-test; **, p<0.01).

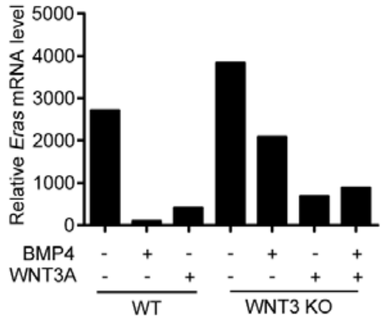

Figure S5. Relative mRNA level of *Eras* in wild type (WT) and *Wnt3* knockout (KO) EpiLCs after BMP4 and/or WNT3A treatment. Data were generated by analyzing GEO dataset GSE49689.

Table S1. Primer sequences for real time RT-PCR

Genes	Primer sequences	Accession No.	Size
Eras	5' GCCTTTGCCCTGCTTGTC 3'<br>5' ACGGCTTTCTGGTGTCGG 3'	NM_181548.2	86 bp
Sox17	5' CTTTATGGTGTGGGCCAAAG 3'<br>5' TTCCAAGACTTGCCTAGCATC 3'	NM_001289464.1	99 bp
Foxf1	5' GCATCCCTCGGTATCACTCAC 3'<br>5' ATCCTCCGCCTGTTGTATGC 3'	NM_010426.2	108 bp
Sox1	5' AGACTTCGAGCCGACAAGAG 3'<br>5' AACTGTGCAAACAGGTGCAG 3'	NM_009233.3	114 bp
Pax6	5' TAACGGAGAAGACTCGGATGAAGC 3'<br>5' CGGGCAAACACATCTGGATAATGG 3'	NM_001244198.1	144 bp
Foxa2	5' CCATCAGCCCCACAAAATG 3'<br>5' CCAAGCTGCCTGGCATG 3'	NM_001291065.1	89 bp
Brachyury (T)	5' CATCGGAACAGCTCTCCAACCTAT 3'<br>5' GTGGGCTGGCGTTATGACTCA 3'	NM_009309.2	136 bp
Flk1	5' GCCCTGCTGTGGTCTCACTAC 3'<br>5' CAAAGCATTGCCCATTCGAT 3'	NM_010612.2	114 bp
Mixl1	5' CGCTCCCTCAGTAACAACGC 3'<br>5' GCTGCCACAGACTTCCAAATG 3'	NM_013729.3	110 bp
Nanog	5' TGCTCCGCTCCATAACTTCG 3'<br>5' GGCTTGTGGGGTGCTAAAAT 3'	NM_028016.3	136 bp
Oct4	5' GAAGCAGAAGAGGATCACCTTG 3'<br>5' TTCTTAAGGCTGAGCTGCAAG 3'	NM_001252452.1	129 bp
Tcf15	5' ATCTGCACCTTCTGTCTCAGC 3'<br>5' GGCTACACCCCTCACTTTCAA 3'	NM_009328.2	87 bp
Tcf7l1	5' GCAGCAAATCCAAGAGGCA 3'<br>5' ACGCAGGGCTATCACAAGG 3'	NM_001079822.2	98bp
Hprt	5' GCCAGTAAAATTAGCAGGTGTTCT 3'<br>5' ATAGGCTCATAGTGCAAATCAAAAG 3'	NM_013556.2	145 bp
